# Autoreactive T Cells in Human Smokers is Predictive of Clinical Outcome

**DOI:** 10.3389/fimmu.2012.00267

**Published:** 2012-08-27

**Authors:** Chuang Xu, Sean Hesselbacher, Chu-Lin Tsai, Ming Shan, Margaret Spitz, Michael Scheurer, Luz Roberts, Sarah Perusich, Nazanin Zarinkamar, Harvey Coxson, Natasha Krowchuk, David B. Corry, Farrah Kheradmand

**Affiliations:** ^1^Baylor College of MedicineHouston, TX, USA; ^2^University of Texas Health Science CenterHouston, TX, USA; ^3^Dan L. Duncan Cancer Center, Baylor College of MedicineHouston, TX, USA; ^4^Michael E. DeBakey VA Medical CenterHouston, TX, USA; ^5^University of British ColumbiaVancouver, BC, Canada

**Keywords:** autoreactive T cells, emphysema, humans, immuno-assays, T cell cloning, tetramers, Th1, Th17

## Abstract

Cross-sectional studies have suggested a role for activation of adaptive immunity in smokers with emphysema, but the clinical application of these findings has not been explored. Here we examined the utility of detecting autoreactive T cells as a screening tool for emphysema in an at-risk population of smokers. We followed 156 former and current (ever)-smokers for 2 years to assess whether peripheral blood CD4 T cell cytokine responses to lung elastin fragments (EFs) could discriminate between those with and without emphysema, and to evaluate the relevance of autoreactive T cells to predict changes during follow-up in lung physiological parameters. Volunteers underwent baseline complete phenotypic assessment with pulmonary function tests, quantitative chest CT, yearly 6-min walk distance (6MWD) testing, and annual measurement of CD4 T cell cytokine responses to EFs. The areas under the receiver operating characteristic curve to predict emphysema for interferon gamma (IFN-γ), and interleukin 6 (IL-6) responses to EFs were 0.81 (95% CI of 0.74–0.88) and 0.79 (95% CI of 0.72–0.86) respectively. We developed a dual cytokine enzyme-linked immunocell spot assay, the γ-6 Spot, using CD4 T cell IFN-γ and IL-6 responses and found that it discriminated emphysema with 90% sensitivity. After adjusting for potential confounders, the presence of autoreactive T cells was predictive of a decrease in 6MWD over 2 years (decline in 6MWD, −19 m per fold change in IFN-γ; *P* = 0.026, and −26 m per fold change in IL-6; *P* = 0.003). In support of the human association studies, we cloned CD4 T cells with characteristic T helper (Th)1 and Th17 responses to EFs in the peripheral blood of ever-smokers with emphysema, confirming antigenicity of lung elastin in this population. These findings collectively suggest that the EF-specific autoreactive CD4 T cell assay, γ-6 Spot, could provide a non-invasive diagnostic tool with potential application to large-scale screening to discriminate emphysema in ever-smokers, and predict early relevant physiological outcomes in those at risk.

## Introduction

Tobacco smoke-induced diseases in humans represent some of the most costly and deadly conundrums facing the world. In human smokers and rodent models of smoke-induced emphysema macrophages followed by neutrophils are recruited to the lungs in large numbers; however, inconsistent with human disease, smoking cessation in mice results in clearance of many if not all inflammatory changes (Guerassimov et al., 2004; Churg et al., 2008). Only after discovery of adaptive immune B and T cells in the lungs of former smokers with emphysema, did it become clear that T cells might be responsible for perpetuating lung inflammation even following smoking cessation in susceptible individuals (Saetta et al., 1998; Grumelli et al., 2004).

CD4 T cells that home to the lung secrete distinct repertoires of cytokines that distinguish them into the T helper type 1 (Th1), Th2, or Th17 cells which are identified by their signature cytokines, namely interferon (IFN)-γ, interleukin (IL)-4, and IL-17 respectively (Del Prete et al., 1993; Kamogawa et al., 1993; Muller et al., 1993; Korn et al., 2009). Recent evidence points to a central role for Th1 and Th17 cell trafficking to the lungs of smokers with emphysema while similar findings have also been described in animal models of smoke-induced emphysema (Harrison et al., 2008; Alcorn et al., 2009; Shan et al., 2009, 2012). What remains unclear is whether specific adaptive immune responses directed against aberrantly exposed antigen(s) could be used to detect human emphysema in the at risk population.

Diseases marked by inflammation of elastin-rich organs such as atherosclerosis of arteries, abdominal aortic aneurysms, and emphysema have been associated with tobacco smoking and the accumulation of inflammatory cells within the affected organs, and concurrently display elevated serum levels of soluble elastin fragments (EFs; Kucich et al., 1991; Rosenbloom et al., 1991; Bizbiz et al., 1997; Lindholt et al., 2001b; Petersen et al., 2002; Galkina and Ley, 2009). We and others have shown that in addition to macrophages and neutrophils that can directly secrete elastin degrading enzymes, emphysematous lung harbors Th1 and Th17 cells that secrete cytokines and chemokines that further enhance the release of matrix metalloproteinases (Grumelli et al., 2004; Freeman et al., 2007; Kelsen et al., 2009; Shan et al., 2009).

Rapid decline in lung function is independently associated with emphysema severity in a subset of susceptible ever-smokers although the underlying mechanisms that drive the pro-inflammatory environment remain unknown (Mohamed Hoesein et al., 2011; Vestbo et al., 2011; Nishimura et al., 2012). The presence of aggregates of T and B cells in the lung parenchyma of smokers that correlated with disease severity indirectly support a role for the adaptive immune system in disease modification (Grumelli et al., 2004; Hogg et al., 2004). Similarly, the marked abundance of IFN-γ and IL-17 secreting T cells in emphysematous lung suggests a pathogenic role for autoimmune mediated inflammation in emphysema (Shan et al., 2009). Nonetheless, the broader physiological implications of elastin-reactive T cells remain undefined and could be explored as a novel diagnostic tool to identify ever-smokers with emphysema. We reasoned that fragments of elastin could provide a source of newly exposed antigen that could perpetuate the recruitment of autoreactive T cells and drive progressive inflammation, as seen in these ever-smokers (Lindholt et al., 2001a; Debret et al., 2005).

In this study we aimed to determine whether detection of highly specific T cell reactivity to EFs could discriminate emphysema, and predict physiological outcome in an at risk population of ever-smokers. In a cross-sectional study we validated that T cell responses to two cytokines, IFN-γ and IL-6 and a newly developed dual cytokine assay (γ-6 Spot), could discriminate emphysema with 90% sensitivity. In a longitudinal study, we found that ever-smokers with autoreactive immune phenotype manifest exaggerated physiological decline over time. Finally we identified smokers that harbor anti-elastin autoimmunity with confirmatory cloning and characterization of CD4 T cells using specific major histocompatibility cluster II (MHC-II) tetramers.

## Materials and Methods

### Study participant phenotypic characteristics

**Figure A1:**
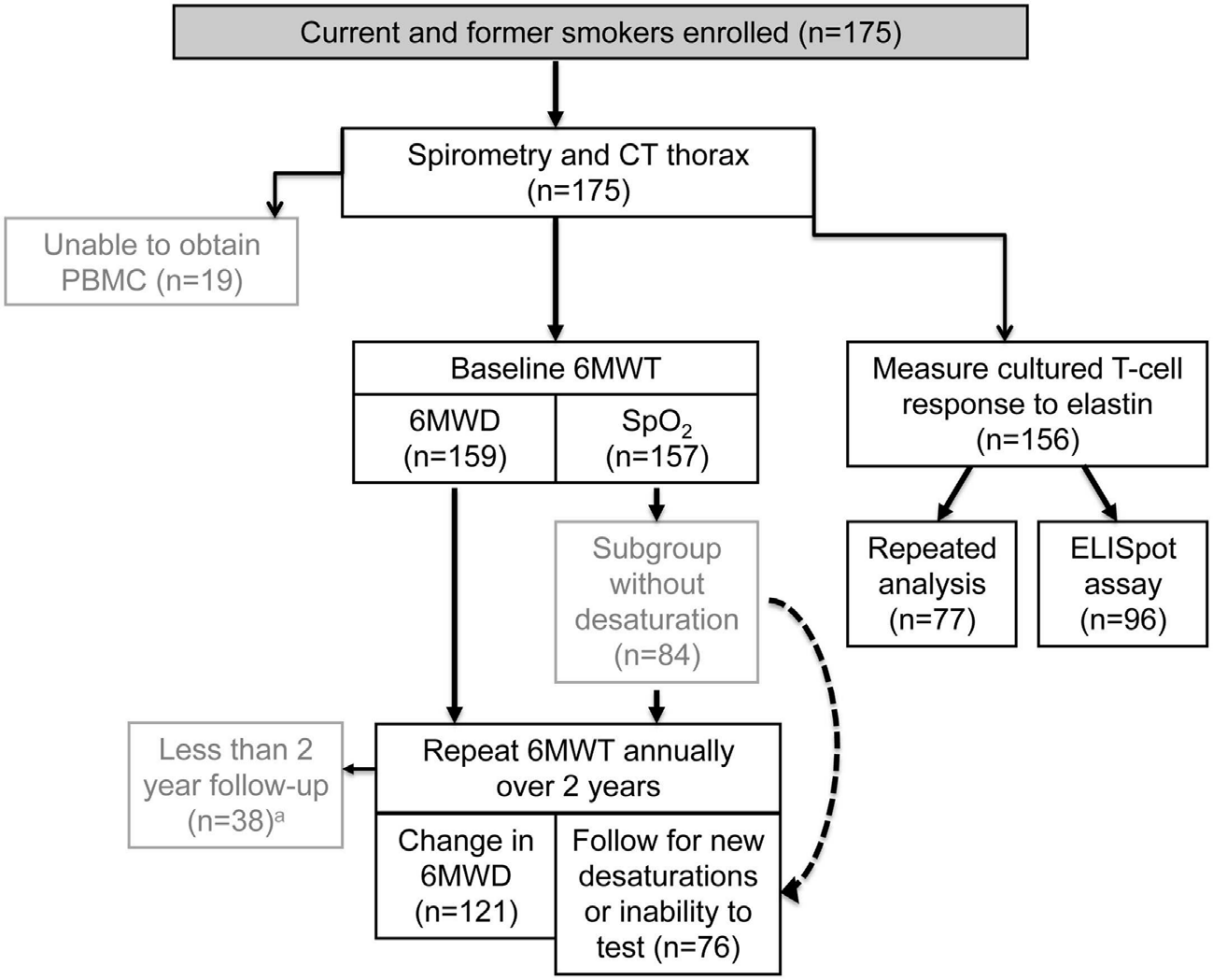
**Schematic overview of the study design**. Abbreviations: CT, computed tomography; 6MWT, 6-min walk test; 6MWD, 6-min walk distance; SpO_2_, peripheral oxyhemoglobin saturation. ^a^Thirty eight subjects did not complete 2-year follow-up of 6MWT: 16 were lost to follow-up or withdrew consent, 9 did not have sufficient time from baseline to complete 2 years, 8 due to illnesses other than respiratory health, 2 due to scheduling conflicts, 2 due to frequent respiratory exacerbations, 1 expired.

We prospectively recruited 175 ever-smokers as part of the Longitudinal Exacerbation Study of Chronic Obstructive Pulmonary Disease (LES-COPD). The demographic and schema of the enrollment and the study population are shown in Table A1 and Figure A1 in Appendix; the clinical characteristics of the volunteers have previously been described (Hesselbacher et al., 2011). Briefly, enrollment criteria included age over 40, no history of concurrent lung cancer, chest surgery, or chronic lung diseases other than COPD (e.g., sarcoidosis, fibrosis, etc.). Participants had no history of allergies or asthma and at the time of initial recruitment had not received oral or systemic corticosteroids during the previous 6 weeks; volunteers were enrolled from three clinics within the Texas Medical Center in Houston, Texas: the Ben Taub General Hospital, the Baylor Clinic, and the Michael E. DeBakey Veterans Administration (VA) hospital. All studies were approved by the Institutional Review Board at Baylor College of Medicine and written informed consent was obtained from all study participants.

### Pulmonary function testing and quantitative chest Computed Tomography

All pulmonary function tests were performed in diagnostic laboratories according to ATS/ERS guidelines (Pellegrino et al., 2005). De-identified CT images were archived onto compact disks, sent to the University of British Columbia (UBC), and were analyzed using the previously validated EmphylxJ custom software (Coxson and Rogers, 2005; Yuan et al., 2009). The CT attenuation of the lung in Hounsfield units (HU) was calculated and used to assess the percentage of emphysema (Coxson et al., 1995, 1999). Percent Low Attenuation Area (%LAA), i.e., attenuation values below −950 HU, of below 7% was used as the non-emphysematous lung; this value has been validated by macroscopic and microscopic evaluation of lung tissue, which found that 6.8% LAA represents the upper limit of non-emphysematous lung (Gevenois et al., 1995, 1996; Yuan et al., 2007, 2009).

### T cell based cytokine assays

Peripheral blood mononuclear cells (PBMCs) were purified using Ficoll-Paque Plus (GE Healthcare) centrifugation technique. CD4^+^ T cells and CD14^+^/CD1a^+^ antigen presenting cells (APCs) were enriched (>90% purity) by magnetic cell sorting (Miltenyi Biotec) from 40 ml of heparinized blood. CD4^+^ T cells (5 × 10^5^) were cultured in the presence of irradiated autologous APC (5 × 10^4^), using a ratio of 1:10 APC to T cells. Duplicate wells were stimulated with EFs (Elastin Products Company, Inc., MO, USA) or were left untreated. The optimal concentration of EFs (30 μg/ml) was determined by dose response studies as previously published and is consistent with the physiological concentration of elastin in plasma (Bizbiz et al., 1997). Alternatively, 300 μM of designed 20-mer overlapping synthesized elastin peptides, or 15-mer elastin peptides identified by prediction algorithms to match DR antigens (PEPscreen, Custom Peptide Libraries; Sigma Genosys) were used to stimulate CD4^+^ T cells *in vitro*. After 3–5 days of co-culture, supernatants were assayed for the presence of IFN-γ, IL-6, IL-10, IL-13, and IL-17 by Luminex assay (Milliplex Human Cytokine kit, Millipore, MA, USA).

The γ-6 Spot assay were set up using ELISpot plates (Millipore) that were incubated overnight at 4°C with 50 μl capture mAb mixture (anti-IFN-γ mAb at 5 mg/ml and anti-IL-6 mAb at 5 mg/ml, eBioscience). CD4^+^ T (2 × 10^5^) cells were isolated from PBMC of emphysema and control volunteers and plated in serial dilution in the presence or absence of 30 μg/ml lung EFs for 24 h. Captured cytokines were visualized as discrete spots by developing with HRP-conjugated anti-IFN-γ- and biotin-conjugated anti-IL-6 antibodies (R&D) and using BCIP/NBT and AEC chromogen (Sigma).

### Reagents, antibodies, and flow cytometry

We designed 20 amino acids (20-mers) peptides, each with 10 overlapping sequences that cover all of the 786 amino acids of human lung elastin (Vrhovski and Weiss, 1998; PEPscreen). Additional antibodies used in these studies were PerCP-conjugated anti-CD4 and anti-HLA-DR antibody (BD Biosciences, San Diego, CA, USA), FITC-conjugated anti-CD44 antibody (eBioscience, San Diego, CA, USA), and anti-CD4 and anti-CD1a conjugated paramagnetic microbeads (Miltenyi Biotec, Auburn, CA, USA).

### CD4^+^ T cell cloning and tetramer staining

Cloning of CD4^+^ T cells using PBMC was accomplished with a modified version of established protocols (Trainor and Morley, 1983; Mariotti and Nisini, 2009). Briefly, PBMC were re-suspended in media (5% human serum) at 1 × 10^6^ cell/ml and were labeled with the intracellular fluorescent dye carboxyfluorescein succinimidyl ester (CFSE). Cells were stimulated with EFs (30 μg/ml; Elastin Products Company, Inc., MO, USA) overnight. CFSE-low populations that divided in response to EFs were isolated using fluorescence activated cell sorting (FACS), distributed in 96-well plates, and stimulated for an additional of 5 days using the same concentration of EFs. Wells were inspected for cell growth and after recombinant human (rh)IL-2 (150 UI/ml; BD Biosciences) stimulation, wells with optimal cell growth were pooled and the T cell blasts were cloned under limiting dilution (0.5 cells/well) in the presence of allogeneic irradiated PBMC (4 × 10^5^ cells/ml), 1 mg/ml phytohemagglutinin-L (Sigma), and 150 IU/ml rhIL-2. After 12–15 days, T cell clones were screened and expanded using lung EFs or specific antigenic peptides that were discovered within individual 20-mer overlapping synthetic peptides. For tetramer staining, T cell clones or PBMC derived T cells were stained with perCP-conjugated anti-CD4 antibody (eBioscience) and APC-conjugated MHC-II tetramers specific for specific elastin molecules (DRB1-0101/LLLLSILHPSRPGGV and DRB1-0101/TGGVPGVGTPAAAAA), as well as a non-specific tetramer hCLIP (DRB1-0101/PVSKMRMATPLLMQA) for 3 h at 37°C. All tetramers were provided by the Tetramer Core Facility of the National Institutes of Health (Emory University, Atlanta, GA, USA). To increase the specificity of elastin-specific T cell clones, a second round of subcloning was performed using limited dilution assay as described above. Specifically, the initial T cell clones were stained with elastin peptide specific tetramer described above, and the tetramer positive T cells were isolated by FACS. Tetramer positive T cells were subsequently cloned under limiting dilution described above and expanded using specific peptide (LLLLSILHPSRPGGV or TGGVPGVGTPAAAAA) with a concentration of 300 μM. The clones were inspected daily and growing clones were harvested after 15 days for further study.

### Six Minute Walk Test: distance walked and oxygen desaturation

Volunteers underwent a baseline and yearly standard 6-min walk test (6MWT) for 2 years (Crapo et al., 2002). Briefly, participants were asked to walk for six consecutive minutes using standardized instructions. Pulse oximetry readings (heart rate and O_2_ saturation) were recorded at the start and every 10 s throughout the walk. Total distance walked in 6-min (6MWD) and the lowest oxyhemoglobin saturation were determined for each test. Oxygen desaturation was defined as a drop to less than 90% O_2_ saturation (Crapo et al., 2002).

### Statistical analysis

Comparisons of two sets of unpaired data were made using Student’s *t*-tests and Mann–Whitney *U* test; paired data were examined using paired *t*-tests or Wilcoxon singed rank tests. Correlations were evaluated by Pearson or Spearman tests, and the discriminatory ability of T cell responses was characterized by area under the **receiver operating characteristics (ROC)** curve (AUC). The optimal cutoff of T cell responses was determined by the maximal Youden index (Youden, 1950). Multivariable linear and negative binomial regression modeling were performed to evaluate the associations of phenotypes with change in 6MWD and exacerbations, respectively. Model variables were selected *a priori* based on medical literature, including age, sex, smoking status (current and former), presence of coronary artery disease, body mass index, and baseline FEV_1_. All analyses were performed using Stata v11.1 software (StataCorp, College Station, TX, USA) or Prism v5.0.2 (GraphPad Software, San Diego, CA, USA). All *P* values are two-sided, with *P* < 0.05 considered statistically significant.

## Results

### CD4^+^ T cells are functionally distinguishable and persist in emphysema

**Figure 1:**
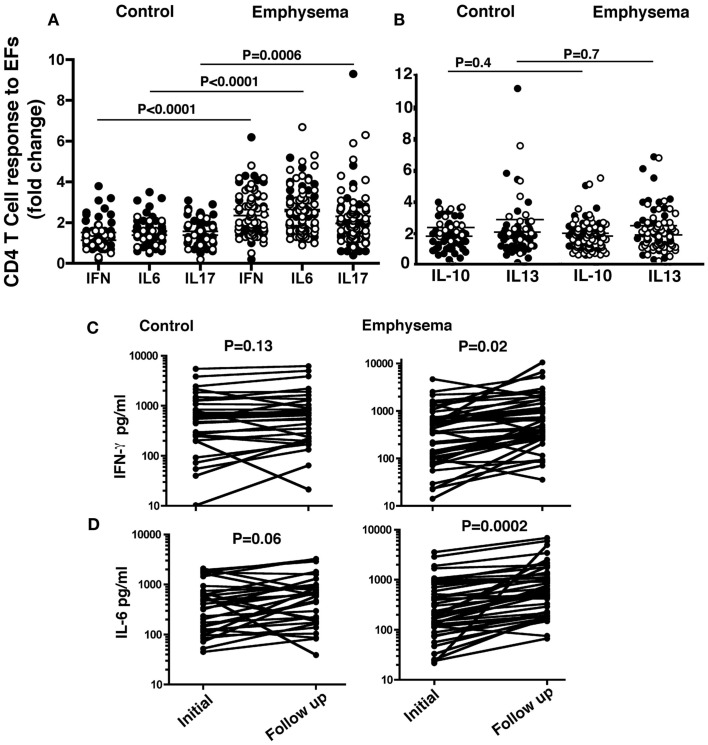
**CD4 T cells are functionally distinguishable and persist in emphysema**. CD4^+^ T cells (5 × 10^5^) and CD14**^+^**/CD1a^+^ APCs (5 × 10^4^) isolated from PBMC of emphysema (*n* = 95) and control (*n* = 61) volunteers were co-cultured (10:1 ratio) for 3–5 days in the presence or absence of lung elastin fragments (30 μg/ml). The mean production IFN-γ, IL-6, and IL-17, **(A)** and IL-10 and IL-13 **(B)** in the supernatants was determined and the data were plotted as fold change over un-stimulated (vehicle) conditions. Each circle represents one volunteer; open circles: former smokers; closed circles: current smokers. Using the same T cell and APC conditions, the concentrations of IFN-γ **(C)** and IL-6 **(D)** were measured from EF-stimulated T cells obtained during a second clinic visit (range 10–24 months following the initial measurement) from emphysema (*n* = 45) and control (*n* = 32) volunteer. *P* values as indicated were determined by the Mann–Whitney test.

We determined whether stimulation of freshly isolated T cells from ever-smokers with or without emphysema with lung-derived EFs resulted in specific Th1 or Th17 cytokine production *in vitro*. Freshly isolated CD4^+^T cells from PBMC of ever-smokers without (controls; *n* = 61) or with emphysema (*n* = 95) that were stimulated with lung EFs showed specific induction of IL-6, IL-17A, and IFN-γ in emphysema (right three scatter plots) as compared to controls (Figure 1A). There were no significant differences in IL-10 or IL-13 production in CD4^+^T cells treated the same way (Figure 1B). Interestingly, former smokers (open circle) with emphysema showed T cell responses to EFs despite reported (average of 12 ± 10 years) smoking cessation.

We next questioned whether elastin-specific T cell responses that we identified at the time of recruitment into the study persist in the same cohort over time. Therefore we measured CD4^+^ T cell cytokine responses to EFs 10–24 months following the first assay in randomly selected ever-smokers with (*n* = 45) or without (*n* = 32) emphysema. We found that there is a large spectrum of the concentration of cytokine secreted by CD4 T cells in response to EFs, but ever-smokers with emphysema largely continued to show robust T cells responses to EFs that both persisted and increased significantly when compared to the initial response (Figures 1C,D). Notably, IFN- γ and IL-6 secretion in response to EFs did not significantly change in the control group, indicating that increases in EF-specific T cell cytokine secretion persist in ever-smokers with emphysema. Although IL-17 response to EFs persisted in the emphysema group, there was no increase in response and as expected, there were no discernable changes in IL-10 or IL-13 responses in either group (data not shown).

### Peripheral blood T cell response to EFs correlates with disease severity

**Figure 2:**
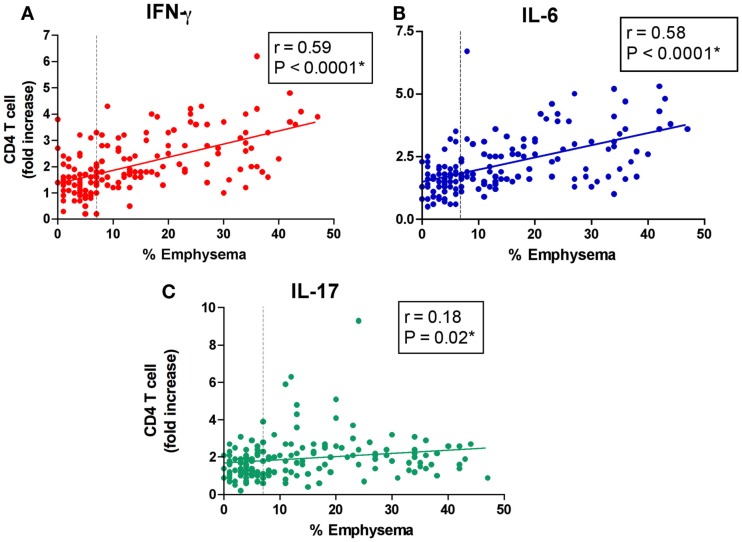
**Peripheral blood T cell response to elastin fragments correlates with disease severity**. The low attenuation area (% emphysema) are plotted against the fold increase in production of **(A)** IFN-γ, **(B)** IL-6, and **(C)** IL-17 by freshly isolated peripheral blood CD4^+^ T cells (*n* = 156) in response to elastin fragments (EFs) compared to nil stimulation as described in Figure 1A. Dashed vertical line (7%) represents the upper limit of normal value for quantification of emphysema.

**Figure A2:**
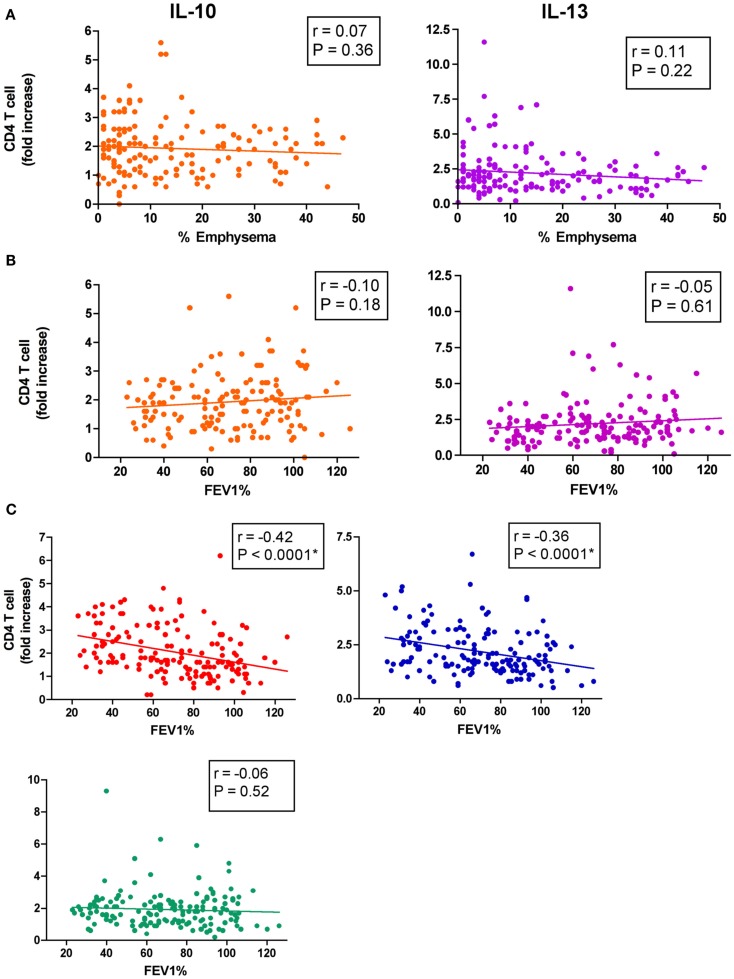
**Cytokine responses to elastin fragments and correlation with disease severity**. **(A)** The correlation between low attenuation area (% emphysema), and **(B)** forced expiratory volume in 1 s % predicted (FEV_1_%) are plotted against the fold increase in production of IL-10, and IL-13 by freshly isolated peripheral blood CD4^+^ T cells (*n* = 112) in response to elastin fragment compared to nil stimulation as described in Figure 1 legend. **(C)** forced expiratory volume in 1 s % predicted (FEV_1_%) are plotted against the fold increase in production of IFN-γ, IL-6, and IL-17 by freshly isolated peripheral blood CD4^+^ T cells (*n* = 156) in response to elastin fragments (EFs) compared to nil stimulation as described above.

We have previously reported a positive correlation between increase in IFN-γ and IL-10, but not IL-4 or IL-13, responses to EFs from CD4^+^ T cells isolated from former smokers with emphysema severity (Lee et al., 2007). Consistently, in this much larger cohort using a CD4^+^ T cells to APCs ratio (10:1), we found a strong positive correlation between the production of IFN-γ (*r* = 0.59, *P* < 0.0001) and IL-6 (*r* = 0.58, *P* < 0.0001), and the severity of emphysema as determined by quantitative CT scan (Figures 2A,B). CD4^+^ T cell secretion of IFN-γ (*r* = −0.42, *P* < 0.0001) and IL-6 (*r* = −0.36, *P* < 0.0001) also showed a significant positive correlation with the severity of airflow obstruction (Figure A2 in Appendix). While IL-17 secretion by CD4^+^ T cells showed a modest correlation with the severity of emphysema, we found no significant correlation with the degree of airflow obstruction as measured by FEV_1_% (Figure 2C, and Figure A2 in Appendix). The production of IL-10 and IL-13 in this large cohort further did not correlate with the severity of emphysema or airway obstruction (Figure A2 in Appendix).

### Dual cytokine (IFN-γ and IL-6) T cell assay detects emphysema: γ-6 Spot Assay

**Figure 3:**
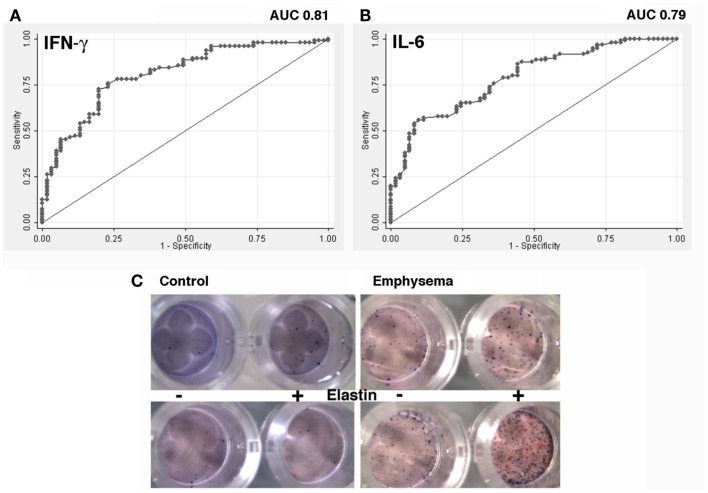
**Discriminatory ability of IFN-γ shown by the receiver operating characteristics (ROC) curve and dual reporter cytokine (IFN-γ and IL-6; γ -6 Spot) assay**. **(A)** Fold change in IFN-γ and **(B)** IL-6 secreted from T cells stimulated with elastin fragments (*n* = 156). The area under the ROC curve for IFN-γ is 0.8085 (95% CI of 0.74–0.88) and for IL-6 is 0.7912 with 95% CI of 0.72–0.86. **(C)** Dual color ELISpot assay showing cytokine producing cells upon elastin stimulation from four representatives. Blue and red spots indicate IL-6 and IFN-γ producing T cells, respectively.

Given that CD4^+^ T cells isolated from peripheral blood samples in ever-smokers with emphysema (irrespective of airflow obstruction) are functionally distinguishable from controls, and the maximal Youden index (Youden, 1950) on the IFN-γ ROC, we defined the threshold for a positive increase in T cell cytokine responses to EFs at 1.5-fold increase over nil stimulation. The area under the AUC for IFN-γ is 0.80 (95% CI of 0.74–0.88) and for IL-6 is 0.79 with 95% CI of 0.72–0.86 (Figures 3A,B). We next examined the number of individuals with or without emphysema with more than a 1.5-fold increase in cytokine, and found that the highest sensitivity (91%) corresponded to either a positive IL-6 or IFN-γ response (Table 1). These data demonstrate that a consistent response could be obtained from analyses of lymphocytes isolated from peripheral blood of ever-smokers. The striking sensitivity of the association between IFN-γ and/or IL-6 responses in T cells stimulated with EFs and emphysema prompted us to develop a PBMC-based assay that could be used as a screening tool to discriminate emphysema in the same cohort. Therefore we next developed an enzyme-linked immunospot (ELISpot) assay that could simultaneously detect T cells expressing IFN-γ and IL-6 (γ-6 Spot) in response to EFs (Figure 3C). Consistent with the cytokine studies we found that the increase in number of IL-6 and IFN-γ-secreting cells in response to 30 μg/ml of EFs showed a similar sensitivity and increased specificity as the cytokine assays (Table 2).

**Table 1:** **Sensitivity and specificity of cytokines in CD4 T cell**.

Cytokine	Control (%LAA < 7) *n* = 61	Emphysema (%LAA > 7) *n* = 95	Specificity (%)	Sensitivity (%)
IFN-γ	23	80	62	84
IL-6	32	84	48	88
IL-17	30	65	51	68
IFN-γ or IL-6	39	86	36	91

**Table 2:** **Sensitivity and specificity of γ-6 spot (ELISpot) assay**.

Cytokine	Emphysema (*n* = 59)	Control (*n* = 37)	Specificity	Sensitivity
IFN-γ	46	8	78	78
IL-6	47	10	73	80
IFN-γ or IL-6	53	12	68	90

### Association between autoreactive T cells and physiological decline

**Figure 4:**
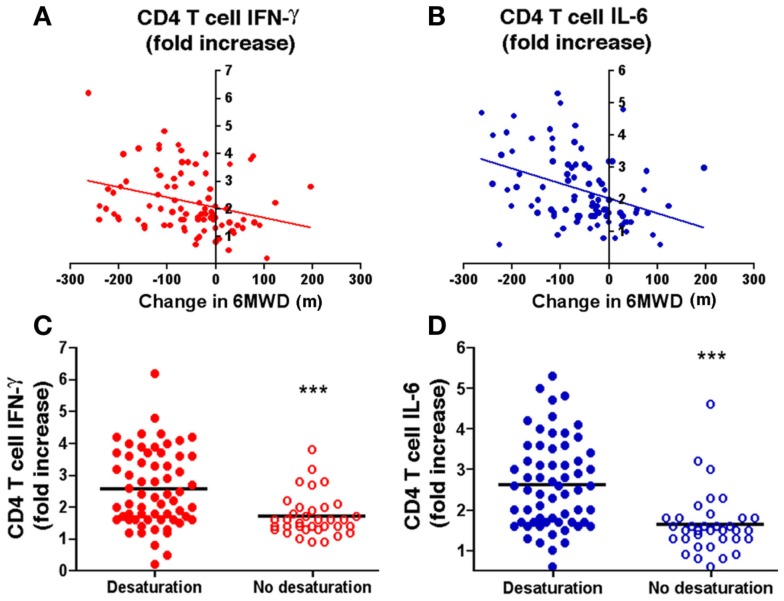
**Longitudinal study of association between autoreactive T cells and physiological decline**. The change in 6-min walk distance (6MWD) in meters over 2 years is plotted against the fold change of CD4^+^ T cells in ever-smokers (*n* = 90) with emphysema **(A)** IFN-γ and **(B)** IL-6 in response to elastin fragments over nil stimulation; *r* = −0.35, *P* = 0.0006 for IFN-γ and *r* = −0.41, *P* < 0.0001 for IL-6). The production of IFN-γ [**(C)**, red] and IL-6 [**(D)**, blue] by peripheral blood CD4^+^ T cells stimulated by elastin fragments is plotted in volunteers that had oxygen desaturation (filled circles, *n* = 62) during the initial or subsequent 6-min walk tests and those that did not (open circles, *n* = 35). Significantly higher levels of IFN-γ (*P* = 0.0002) and IL-6 (*P* < 0.0001) were measured in volunteers with oxygen desaturation.

**Figure A3:**
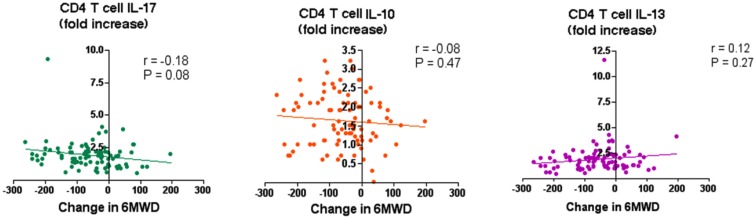
**Longitudinal study of association between IL-17, IL-10, and IL-13 in autoreactive T cells and physiological decline**. The change in 6-min walk distance (6MWD) in meters, over 2 years is plotted against the fold change of CD4^+^ T cells in ever-smokes (*n* = 90) in IL-17, IL-10, and IL-13 in response to elastin fragments over nil stimulation. No significant associations were found among these three cytokines and changes detected in 6MWD.

The findings of elastin-specific T cells and autoreactive responses to EFs strongly suggested a possible relationship between disease progression as assessed by physiological decline and persistent systemic autoimmune inflammation in ever-smokers. Because smoking related decline in lung function is quite variable and thus a relatively unreliable measurement of physiological decline, instead we opted to use a measurement of physical activity, the 6-min walk distance (6MWD) test (Figure A1 in Appendix). Univariate analyses of the 6MWD (in meters) and secreted T cell IFN-γ and IL-6 showed a strong inverse association over 2 years (Figures 4A,B). Multivariate analyses adjusting for potential confounders, including age, sex, smoking status, baseline FEV_1_, coronary artery disease, and body mass index, confirmed that the associations remained significant (decline in 6MWD, −19 m per fold change in IFN-γ; *P* = 0.026, and, −26 m per fold change in IL-6; *P* = 0.003). There was no significant association between 6MWD and fold change in IL-17, IL-10, or IL-13 (Figure A3 in Appendix). In support of the observed decline in physiological capacity, we also found that individuals with reduced oxygen saturation during annual 6MWT showed significantly greater CD4^+^ T cell response to EFs as evidenced by higher IFN-γ (*P* = 0.0002) and IL-6 (*P* < 0.0001) fold induction when compared to those without reduced oxygen saturation (Figures 4C,D). Multivariate analyses revealed no associations between cytokine production and outpatient respiratory infections, COPD-related hospitalizations, or non-COPD hospitalizations (data not shown). Thus, elastin-specific autoimmune reactivity can be detected by standard immunological assays and persistence of autoreactive T cells in peripheral blood portends disease progression as assessed by decreases in relevant functional parameters.

### Identification of elastin-specific T cell epitopes

**Figure 5:**
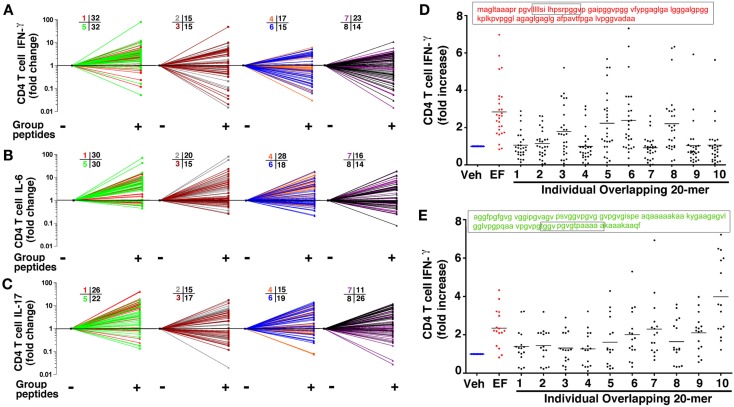
**Overlapping elastin peptides contain multiple T cell epitopes**. Seventy eight overlapping peptides 20 amino acids (20-mer) in length spanning human elastin were synthesized and pooled into 8 groups designated with eight different colors (described in Figure A4 in Appendix). CD4^+^ T cells (5 × 10^5^) were co-cultured with CD14^+^/CD1a^+^(5 × 10^4^) autologous APCs in the presence or absence of 20-mers (300 μM) in each group. Fold increase over nil stimulation in secreted IFN-γ **(A)** IL-6 **(B)** and IL-17 **(C)** are shown in 2 combined groups per spray plot; *n* = 39. The numbers corresponding to each color indicate the numbers of individuals with a positive (>1.5-fold increase) response over nil stimulation. Using the same T cell/APC assay, IFN-γ fold increase induced by each 20-mer (1–10) within **(D)** group 1 (shown in red) and **(E)** group 5 (shown in green) were plotted as fold increase relative to nil stimulation; each dot represent one volunteer; *n* = 27 for group 1, *n* = 16 for group 5. Lung elastin fragments (EFs; 30 μg/ml) were used as positive control. Veh, vehicle control.

**Figure A4:**
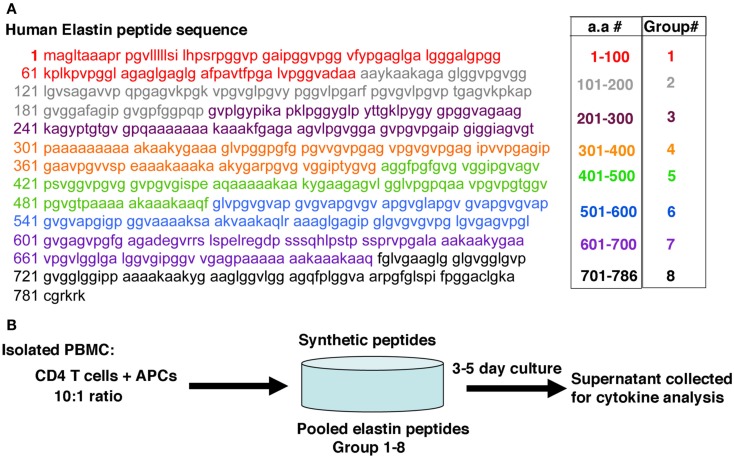
**Schematic representation of the 20-mer elastin peptides and pooled groups**. **(A)** Twenty amino acid (20-mer) peptides were synthesized that span the entire human elastin molecule and were grouped into 8 pools. **(B)** Each pooled groups were added to CD4^+^ T cell (5 × 10^5^) and CD1a^+^ APCs (5 × 10^4^) for 3–5 days. Supernatant were collected and stored at −80 for batch analysis of selected cytokines using LINCOplex assay.

**Figure A5:**
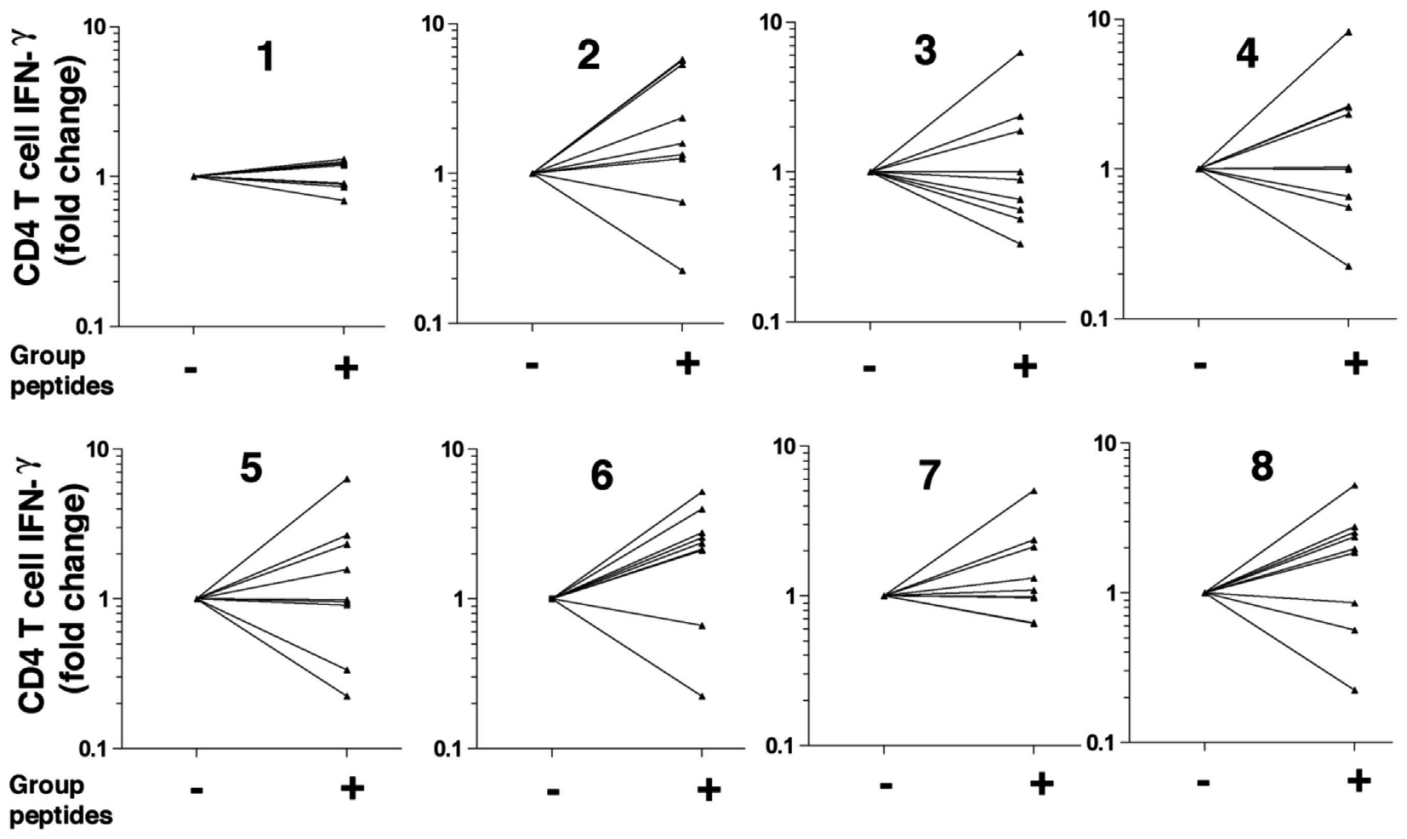
**Spray plot of cytokines secreted in T cells isolated from ever-smoker controls**. Seventy eight overlapping peptides 20 amino acids (20-mer) in length spanning human elastin were synthesized and pooled into 8 groups designated with eight different colors (described in Figure A4 in Appendix). CD4^+^ T cells (5 × 10^5^) were co-cultured with CD1a^+^ (5 × 10^4^) APCs isolated in control ever-smokers without emphysema in the presence or absence of 20-mers (300 μg/ml) in each group. Fold increase over nil stimulation in secreted IFN-γ are shown in 2 combined groups per spray plot; *n* = 9. Numbers on top of each spray plot represents the specific pooled group as described in Figure A4 in Appendix).

The preceding findings corroborate that elastin-specific Th1 and Th17 cells persist in peripheral blood of ever-smokers with emphysema. Although the findings are highly specific, they were derived using commercially available, human lung EFs that are created using neutrophil elastase digestion (Starcher and Galione, 1976). Thus, to explore the immunogenic nature of elastin, we synthesized 78 overlapping 20 amino acid (20-mers) long peptides that span the entire length of the molecule (Figure A4 in Appendix). In the first screen of peptides that were pooled into 8 groups, as depicted using spray plots, we found that peptides in groups 1 and 5, shown in red and green, elicited the most significant and consistent increases (at least 50% over vehicle control) in secretion of IFN-γ, IL-6, IL-17A from CD4^+^ T cells when compared to other peptide pools (e.g., groups 2–4, and 6–8; Figure 5). These findings were specific to ever-smokers with emphysema because examination of CD4^+^ T cells in controls (ever-smokers without emphysema) with positive T cell responses to EFs of greater than 50% increase, did not identify the same regions within the peptide sequences (Figure A5 in Appendix). The existence of many potential T cell elastin epitopes is suggested by the positive T cell responses in all peptide pools that we tested (Figures 5A–C). However, to further pinpoint the specific epitopes and because of the consistently higher responses seen in groups 1 and 5, CD4^+^ T cells from those with emphysema were subsequently stimulated with individual overlapping 20-mer peptides within the amino acids 1–100 and 401–500. As expected, many, but not all, peptide sequences in these two pools elicited significant increases in IFN-γ secretion from CD4 T cells (Figures 5D,E). These findings confirm that elastin epitope-specific Th1 and Th17 cells are selectively present in the peripheral blood of ever-smokers with emphysema.

### CD4^+^T cell clones respond to elastin fragments

**Figure 6:**
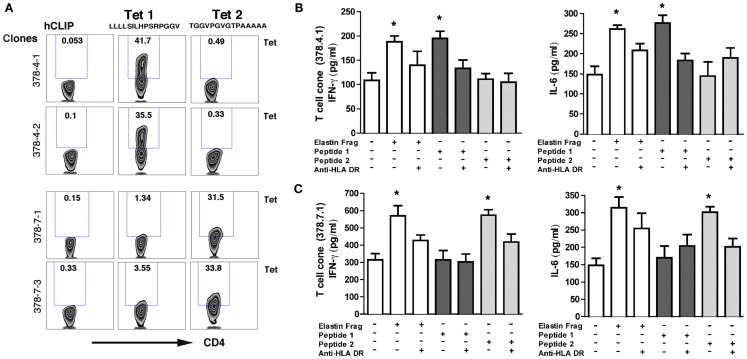
**Cloning and characterization of EF-specific CD4 T cells**. **(A)** Representative flow cytometry plot for two different CD4^+^ T cell clones that were stained with antibody to CD4 (perCP-conjugated) and APC- conjugated MHC-II tetramers specific for elastin molecule (DRB1-0101/LLLLSILHPSRPGGV and DRB1-0101/TGGVPGVGTPAAAAA), as well as a non-specific tetramer hCLIP (DRB1-0101/PVSKMRMATPLLMQA) are shown. Relative % abundance of tetramer positive CD4^+^ T cells is shown above each of the gated area in the plots. Data are representative of three independent assays. Representative measurement of IL-6 secretion in T cell clones **(B,C)** in response to peptide 1 and peptide 2 (300 μM each) as described in experimental condition above **(A)**. Data are mean ± s.e.m. of three measurements, represents one of six independent studies. *P < 0.05; determined by the Student’s *t*-test comparing to the baseline T cell cytokine measurement.

We next used PBMCs that were isolated from participants with the most robust immune responses to EFs to clone elastin-specific T cells. Four of the eight volunteers that we studied yielded multiple elastin-specific CD4^+^ T cell clones that responded to EFs with greater than 50% increase in IFN-γ and IL-6 secretion (data not shown). Based on the *in vitro* T cell activation studies using synthetic 20-mer overlapping elastin peptides, we searched prediction engines^1^^,^^2^ to find sequences known to bind a common class II MHC molecule (DRB1) with high affinity and found three putative 15-mer peptide sequences. We designed and synthesized two peptides that induced the strongest cognate cytokine secretion in T cells and had the highest predicted binding scores, belonging to group 1 and group 5 peptides designated as peptides 1 (LLLLSILHPSRPGGV) and peptide 2 (TGGVPGVGTPAAAAA), respectively. We next isolated T cells from the peripheral blood of patients with a strong cytokine response to elastin stimulation using cells labeled with the intracellular fluorescent dye CFSE. T cells with low CFSE were isolated with a flow sorter and were stained with two MHC-II tetramers using the same identified immunodominant elastin peptide 1 and peptide 2 that we had used to validate their immunogenic properties. We found tetramer positive staining in several cloned T cells for one or both tetramers (Figure 6A, and data not shown), therefore to increase the purity of T cells responding to elastin, we sorted tetramer positive T cells and performed a second round of T cell cloning using limiting dilution technique (Trainor and Morley, 1983). Consistently, a CD4^+^ T cell clone (e.g., 378-4-1) with over 40% detectable tetramer 1 staining secreted higher concentration of IL-6 and IFN-γ in response to elastin peptide 1, while no significant response was detected with peptide 2 stimulation under the same conditions (Figures 6A,B). Similarly, tetramer 2 staining was detected in over 30% of T cell clones (e.g., 378-7-1) that specifically responded to peptide 2, but not peptide 1 (Figures 6A,C). Further, anti-DR blocking antibodies either partially or fully inhibited IL-6 and IFN-γ secretion, indicating specific MHC-II dependent antigen responses to peptides 1 and 2 (Figures 6B,C).

**Figure 7:**
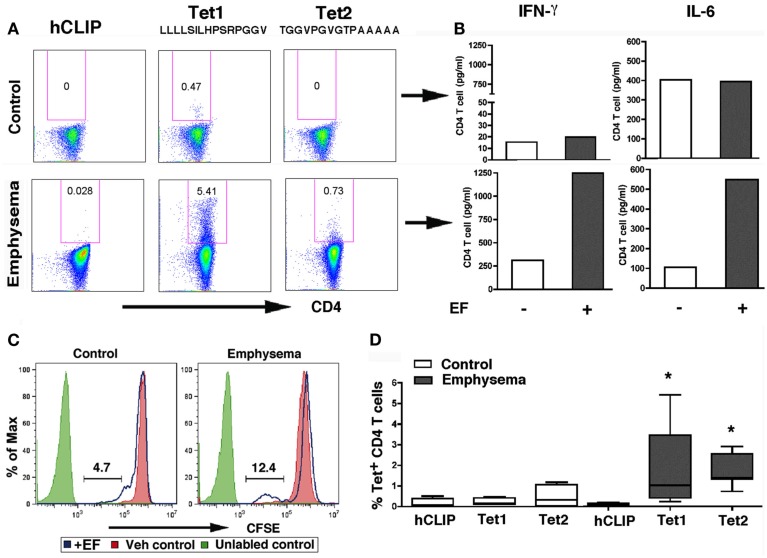
**Increased relative abundance of elastin positive tetramers in emphysema**. **(A)** Representative flow plots of controls (*n* = 5) and emphysema (*n* = 7) cases for freshly isolated CD4^+^ T cells from control and emphysema that were stimulated with elastin fragments (EFs) for 3 days and were stained with elastin-specific and control tetramers as described in Figure 6C. Relative % abundance of tetramer positive CD4^+^ T cells is shown above each of the gated area in the plot. **(B)** IFN- γ and IL-6 fold change in response to elastin fragment (30 μg/ml) over nil stimulation for the corresponding patients. Cytokine concentrations were measured by LINCOplex. **(C)** Representative histograms of CD4^+^ T cells CFSE-labeled isolated from PBMC of control or emphysema volunteers cultured overnight in the presence (blue lines) or absence (red filled lines) of elastin fragments (30 μg/ml). Unlabeled control cells are shown in solid green lines. **(D)** Cumulative relative % abundance of tetramer positive CD4^+^ T cells in controls (*n* = 5) and emphysema (*n* = 7) for tetramer 1 and 2 as well as a non-specific tetramer hCLIP as described in Figure 6A.

**Figure A6:**
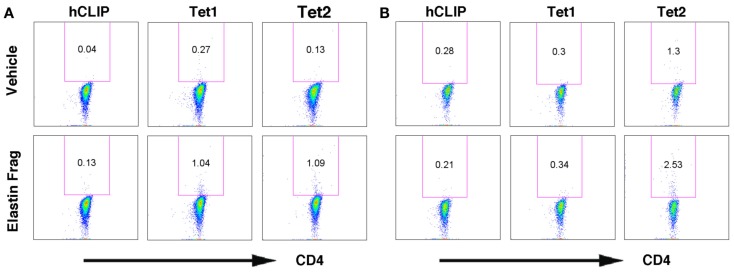
**Elastin fragments increases relative abundance of % Tetramer positive CD4 T cells**. Flow cytometry plot for freshly isolated CD4 T cells that were stimulated without (vehicle) or with elastin fragments (EFs; 30 μg/ml) stained with antibody to CD4 (perCP-conjugated) and APC-conjugated MHC-II tetramers specific for elastin molecule (DRB1-0101/LLLLSILHPSRPGGV and DRB1-0101/TGGVPGVGTPAAAAA), as well as a non-specific tetramer hCLIP (DRB1-0101/PVSKMRMATPLLMQA) are shown. Relative % abundance of tetramer positive CD4^+^ T cells is shown above each of the gated area in the plots. Representative of a subject showing **(A)** approximately 10-fold increase or **(B)** moderate increase in relative % abundance of Tet1 and Tet 2 in response to EFs stimulation. Data are representative of three independent assays.

To confirm the presence of autoreactive elastin-specific T cells, we used freshly isolated CD4^+^ T cells from control and emphysema volunteers and determined the relative abundance of elastin tetramer positive T cells. We found that while some CD4^+^ T cells in emphysema had increased relative abundance of elastin positive tetramers without any stimulation, in most cases, up to 10-fold increase in T cell binding to the tetramers was found following 3 days of T cell stimulation with EFs (Figures 7A,B and Figure A6 in Appendix). Therefore, we assessed the relative abundance of tetramer positive CD4^+^ T cells following 3 days of culture with EFs in controls and emphysema volunteers. We found a higher proliferation response, and a larger relative abundance of tetramer positive CD4^+^ T cells when compared to controls (Figures 7A–D). The same individuals showed significantly increased T cell cytokine responses to EFs, confirming that the pathogenic T cells secrete IFN-γ and IL-6 in an antigen-specific manner (Figure 7B). Notably, the same T cells failed to secrete IL-13 and IL-10 under the same conditions (data not shown).

## Discussion

In this comprehensive study, we report the first description of a screening assay designed to discriminate emphysema and provide the results of a longitudinal study that links T cell immune profiles with physiological decline among ever-smokers. Using emphysema as the main hallmark of smoking-induced lung damage, peripheral blood CD4^+^ T cell immune responses to elastin consistently discriminated those with and without disease over 2 years of follow-up. Furthermore, we discovered that the discriminatory T cell cytokine signature consisted of IFN-γ and IL-6, and to a lesser extent IL-17A. EF-specific induction of these cytokines was significantly increased in those with emphysema irrespective of smoking status and baseline FEV1 (both were controlled for in multivariate analyses), and was associated with a rapidly progressive decline in physiological function.

Several important findings emerge from our investigations and description of the autoreactive T cells screen assay that discriminate emphysema in ever-smokers. First, using the host immune response, we addressed the longstanding question regarding the remarkable heterogeneity seen in disease expression and progression in smokers with similar smoking histories that typically spans a spectrum ranging from no disease to the most severe and debilitating forms of lung destruction (Agusti et al., 2010; Han et al., 2010). Our findings here may shed new light on this well known clinical observation, as evidenced by the fact that not all ever-smokers develop autoreactive T cells. While many genetic factors shape individual responses to environmental insults such as cigarette smoke, our findings here point to the need to define the genetic basis of elastin autoimmunity induced by exposure to cigarette smoke (Kheradmand et al., 2012).

Detection of autoreactive T cells and its inverse association with accelerated physiological decline, as we show here, supports the hypothesis that T cells responses to elastin might be a poor prognostic indicator in a subset of ever-smokers. In addition to conventional T cell restimulation assays used to determine the presence of elastin-specific immunity, such prognostic studies could also include the use of tetramer technology to more rapidly detect and quantify elastin-specific T cells, as we applied for the first time here. Identification of the immunodominant peptides within EFs provides the exciting possibility that autoreactive cells could be suppressed using alteration of the immune response, thus providing a uniquely specific therapy for emphysema. Further long-term and independent cohort studies are required to establish the feasibility of the prognostic and therapeutic strategies suggested by our findings.

Unlike the well-accepted Global initiative on Obstructive Lung Disease (GOLD) staging system that defines specific COPD strata based on measurements of airflow obstruction, there exists no similar classification of emphysema (GOLD Executive Committee, 2008). Because the aim of our investigation was to determine whether autoimmune T cells in lifetime ever-smokers could discriminate lung parenchymal disease, we used CT-based quantitation of emphysema and not pulmonary function-based analyses of airflow obstruction (Gevenois et al., 1995, 1996; Yuan et al., 2007, 2009). Importantly, our findings demonstrate that, irrespective of lung function status, the presence of elastin-specific CD4^+^ T cells in the peripheral blood correlates with the degree of emphysema. Airflow limitation is not an inevitable consequence of emphysema and, indeed, the mechanisms governing reduced airflow in emphysema are not well understood. Not surprisingly, therefore, attempts to use indices of lung function to predict clinically relevant outcomes in smokers have shown mixed success (Highland et al., 2003; Lapperre et al., 2009; Han et al., 2010). In contrast, our ability to correlate elastin-specific T cell responses in smokers to declines in functional performance in the 6MWT point to the fundamental importance of autoimmunity in lung disease in smokers.

The importance of IFN-γ and IL-17A in autoimmunity has been established in both humans and animal models (Littman and Rudensky, 2010). Neurodegeneration in multiple sclerosis, synovial inflammation in rheumatoid arthritis, inflammatory bowl disease, and type I diabetes have all been shown to be in part mediated by IL-17A and IFN-γ (Toh and Miossec, 2007; Yamada et al., 2008; Uhlig and Powrie, 2009; Durant et al., 2010; Kaser et al., 2010). Moreover, the harmful role of IL-17 in autoinflammatory diseases has been defined through the beneficial effect of neutralization of this cytokine in clinical studies involving autoimmune disorders (Hueber et al., 2010). Therefore, given the autoimmune nature of emphysema, our human translational studies suggest that therapies that neutralize IL-17, IFN-γ, and related cytokines in ever-smokers with emphysema manifesting increased T cell responses to EFs might be similarly effective. While the exact contribution of these cytokines in progression of autoimmune diseases is an intense area of investigation, multiple susceptibility loci have been associated with the reduction of immune tolerance to self-antigens that most likely drive development of autoreactive T cells (Lettre and Rioux, 2008).

In addition to increased secretion of IFN-γ and IL-17 from autoreactive T cells, we have also identified IL-6, a pleotropic cytokine that together with TGF-β is required for differentiation of native T cells into pathogenic Th17 cells (Chang and Dong, 2011). Human studies have shown increased IL-6 serum concentrations with age that associate with specific single nucleotide polymorphisms (SNPs) in the IL-6 gene, but there was no evidence found for an association between common variations in this gene and adverse health outcomes in a population of older adults (Walston et al., 2007). While a retrospective study using two large cohorts identified three IL-6 SNPs that are associated with a rapid decline in lung function (He et al., 2009), it should be noted that none of the haplotypes were associated with an increase in basal levels of IL-6, suggesting that the identified SNPs are most likely not a single determinant of basal secretion of serum IL-6. While we did not evaluate the basal concentration of any cytokine in the serum of our cohort, we found that there was a large variability in the baseline secretion of IL-6 from T cells that did not discriminate ever-smokers with or without emphysema (data not shown); however we found that T cell responses to EFs resulted in a significant and sustained increase in IL-6 secretion.

The immune profile in those with emphysema may be distinct from those with predominant airway obstruction because, while they were both associated with the production of IFN-γ and IL-6 in response to EFs, only emphysema severity but not airway obstruction correlated with the magnitude of IL-17 production. Thus, production of IL-6 and IL-17 in response to lung antigen is seen in ever-smokers with emphysema, but IFN-γ is produced most consistently in those with both airflow obstruction and emphysema, possibly because these smokers tend to have more severe disease.

In parallel to the possible role of autoimmune mediated recruitment of inflammatory cells to the lungs, proteolytic cleavage of matrix molecules has been shown to provide the necessary environmental factors associated with human COPD (Senior et al., 1984; van Houwelingen et al., 2008). In particular, collagen degradation can elaborates *N*-acetyl Pro-Gly-Pro (PGP) tripeptides that act as a potent neutrophil chemoattractants *in vivo*, a process that is mediated through activation of CXCR2 (Weathington et al., 2006). Our findings demonstrate that in addition to the role of PGPs, the presence of elastin autoreactive T cells in peripheral blood portend disease progression over time.

The observational nature of this study makes it impossible to determine a causal relationship between phenotype in ever-smokers and the associated clinical impact noted. Furthermore, because of the significant difference in the elastin composition of the lungs in human (24%) and rodents (2.4%; Starcher and Galione, 1976), smoke-induced lung disease in inbred strains of mice most likely will not provide the exact immunological features as those found in humans with emphysema. Nonetheless, identifying the factors that specifically lead to destructive changes in human emphysema and in animal models of smoke-induced emphysema, are needed for understanding the pathogenesis of a potentially disabling and fatal response to cigarette smoke.

## Conflict of Interest Statement

The authors declare that the research was conducted in the absence of any commercial or financial relationships that could be construed as a potential conflict of interest.

## Figures and Tables

**Table A1:** **Clinical and demographic information of the study participants**.

Group	Controls	Emphysema
Number	61	95
Age, mean (±s.d.)	55 ± 9	62 ± 9
Height, cm (±s.d.)	170 ± 10	175 ± 8
Weight, kg (±s.d.)	83 ± 18	87 ± 20
BMI, kg/m^2^ (±s.d.)	29 ± 6	28 ± 7
Male, no. (%)	31 (51%)	79 (83%)
**ETHNICITY, NO. (%)**		
Black	32 (52%)	29 (31%)
Hispanic	3 (5%)	4 (4%)
White	25 (41%)	61 (64%)
Other	1 (2%)	1 (1%)
**SMOKING STATUS, NO. (%)**		
Current	46 (75%)	38 (40%)
Former	15 (25%)	57 (60%)
Never	0 (0%)	0 (0%)
Pack-years, mean (±s.d.)	47 ± 41	58 ± 37
Quitting years, mean (±s.d.)	13 ± 14	11 ± 10
**LUNG FUNCTION**		
% FEV1 (± s.d.)	83 ± 19	62 ± 25
FEV1/FVC ratio (±s.d.)	73 ± 10	54 ± 16
%LAA (±s.d.)	3 ± 2	21 ± 11

^*^s.d., Standard deviation; cm, centimeters; kg, kilograms; BMI, body mass index; no., number; %FEV1, forced expiratory volume in 1 s, percent of predicted; FVC, forced vital capacity; pack-years, number of years smoked × packs per day; quitting years, number of years since last reported smoking activity; %LAA, % low attenuation area (−950 HU) by CT quantification.

## Footnotes

^1^www-bimas.cit.nih.gov

^2^www.syfpeithi.de/
